# Extraoral Retrograde Root Canal Filling of an Orthodontic-induced External Root Resorption Using CEM Cement 

**Published:** 2014-03-08

**Authors:** Sanam Kheirieh, Mahta Fazlyab, Hassan Torabzadeh, Mohamad Jafar Eghbal

**Affiliations:** a * Iranian Center for Endodontic Research, Research Institute of Dental sciences, Shahid Beheshti University of Medical Sciences, Tehran, Iran;*; b * Dental Research Center, Research Institute of Dental sciences, Shahid Beheshti University of Medical Sciences, Tehran, Iran*

**Keywords:** Calcium Enriched Mixture, CEM Cement, Endodontics, External Inflammatory Root Resorption, Intentional Replantation, Oral Surgery, Orthodontics, Root Resorption

## Abstract

Inflammatory external root resorption (IERR) after orthodontic treatments is an unusual complication. This case report describes a non-vital maxillary premolar with symptomatic extensive IERR (with a crown/root ratio of 1:1) after receiving orthodontic treatment. The first appointment included drainage, chemo-mechanical preparation of the canal and intra-canal medication with calcium hydroxide (CH) along with prescription of analgesic/antibiotic. The subsequent one-week follow-up revealed the persistence of symptoms and formation of a sinus tract. Finally, extraoral endodontic treatment was planned; the tooth was atraumatically extracted and retrograde root canal filling with calcium enriched mixture (CEM) cement was placed followed by tooth replantation. Clinical signs/symptoms subsided during 7 days postoperatively. The sinus tract also resolved after one week. Six-month and one-year follow-ups revealed complete healing and a fully functional asymptomatic tooth. This case study showed favorable outcomes in a refractory periapical lesion associated with orthodontically induced extensive IERR. The chemical as well as biological properties of CEM cement may be a suitable endodontic biomaterial for these cases.

## Introduction

Inflammatory external root resorption (IERR), is one of the complications most frequently associated with inflammation of the periradicular tissues stemming from the microorganism-induced intracanal infection; which mostly leads to progressive damage of root structure and finally tooth loss [1]. Other common etiologic factors, which have been identified for IERR, are apical periodontitis, orthodontic tooth movement, traumatic injuries, expanding tumors and cysts, internal bleaching and idiopathic factors. The biomechanisms of IERR depend on injury and different stimulations for instance the mechanical damages following intensive orthodontic forces which could result in pulp necrosis [2]; which by itself can enclose additional egression of bacterial endotoxin from infected necrotic pulp space. Along with the process of IERR, the apical periodontitis lesion progressively expands. 

Experts are of the opinion that vital teeth with orthodontic induced IERR do not need to undergo endodontic therapy, as this approach does not eliminate the etiologic factor of the condition [3]. However, in teeth with necrotic pulps, endodontic intervention may be required. In such cases, common treatment protocol for progressive IERR comprises of chemomechanical disinfection of the root canals using intra-appointment irrigants like NaOCl and inter-appointment dressing of the root canal system with calcium hydroxide (CH) to provide an alkaline pH both for inactivation of odontoclasts and for bacterial elimination and neutralizing their byproducts such as endotoxin [4]. 

Extraoral endodontic treatment (EET) may be performed with a retrograde approach, which includes cleaning/shaping of the root canal system and filling the prepared root-end with appropriate biomaterial. For this purpose, different materials such as amalgam, gutta-percha, zinc oxide-eugenol cements, glass ionomer cement, gold foil pellets, Cavit, composite resin, mineral trioxide aggregate (MTA) and calcium enriched mixture (CEM) cement have been used [5, 6]; among which, MTA and CEM cement are the unique biomaterials with proved cementogenic/osteogenic activity in histological evaluation [7, 8].

**Figure 1:**
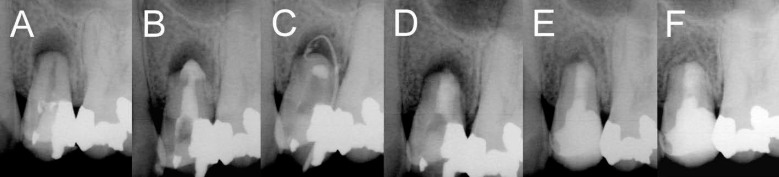
*A)* Extensive IERR associated with a periapical lesion; *B)* Placing inter-appointment calcium hydroxide (CH) dressing after drainage; *C)* Formation of a sinus tract after one week; *D)* Radiographic confirmation of accurate tooth replantation after extraoral retrograde root canal filling with CEM cement; *E-F)* Radiographic healing and formation of new bone after six months *(E)* and 1 year *(F)* follow-ups

This case report represents the diagnosis and treatment challenges of an EIRR lesion around a symptomatic non-vital maxillary premolar, which had previously been under orthodontic treatment; also extraoral endodontic treatment of this tooth using CEM cement and up to one-year follow-up results are presented.

## Case report

A 22 year-old female with a symptomatic maxillary left second premolar was referred to a private endodontic clinic by her orthodontist three months after completion of orthodontic treatment. The patient’s medical history was noncontributory and her body temperature was 39.5^º^ C. Intraoral examination revealed a diffused swelling in the apical area of the involved tooth, class II tooth mobility and severe tenderness to percussion and palpation; periodontal probing was within the normal range (<3mm). Cold test with Endo-Frost cold spray (Roeko, Langenau, Germany) elicited no response in involved tooth; the adjacent teeth revealed a positive response to cold test. The periapical x-ray revealed a deep restoration adjacent to the pulp and a large radiolucent periradicular lesion surrounding the open-apex and resorbed root (Figure 1A), which was assumed to be due to pulp necrosis and peri-radicular inflammation and the intensive orthodontic forces acting as boosting factors. The final diagnosis for the tooth was pulpal necrosis with acute apical abscess along with inflammatory external root resorption. Based on clinical findings, the first treatment option was drainage via the access cavity followed by multi-visit orthograde endodontic treatment and placement of an apical plug. After complete explanation of the procedures and risks/benefits for the patient, an informed consent was obtained. 

After a 0.2 % chlorhexidine mouth rinse and administration of local anesthesia using 2% lidocaine with 1:80000 epinephrine (Daroupakhsh, Tehran, Iran), the involved tooth was isolated and the access cavity was prepared. On entering the pulp chamber, a purulent drainage was observed, and patient’s tenderness immediately subsided. After radiographic working length determination, cleaning and shaping of the root canal was performed; the root canal was irrigated and filled with 5.25% sodium hypochlorite for 15 min along with passive instrumentation. CH powder (Golchay, Tehran, Iran) and normal saline were mixed to prepare a creamy paste which was then placed within the root canal after canal drying with large paper points (Figure 1B). A *TID* prescription of 500 mg Amoxicillin capsules for 7 days and by-the-clock assumption of 400 mg ibuprofen supplemented the treatment. The patient was recalled one week after operation; the patient had a draining sinus tract (Figure 1C) and class III tooth mobility was observed. Since the patient refused tooth extraction/implant treatment and due to remained clinical sign/symptoms of mobile short-rooted tooth, we modified the treatment plan to EET. 

**Figure 2:**
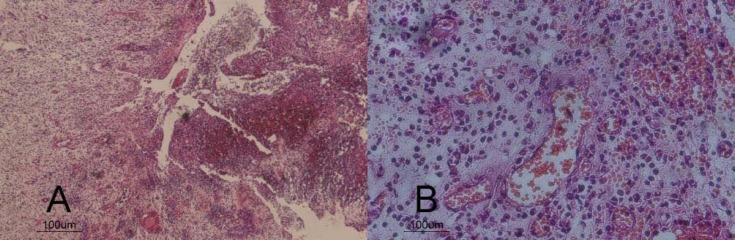
Histological image of periapical granulomatous lesion of the tooth with two magnifications

After administering local anesthesia via infiltration of 2% lidocaine with adrenalin 1:80000, the tooth was atraumatically extracted; meanwhile the apical lesion was curetted and sent for histopathological examination. While holding the tooth in a forceps, the root canal was retrogradely prepared using Peeso reamers #2 (Dentsply, Maillefer, Ballaigues, Switzerland) and copious saline irrigation; finally the cavity was dried with sterilized paper points. Then CEM cement powder and liquid (BioniqueDent, Tehran, Iran) was mixed according to the manufacturer’s instructions. CEM was retrogradely inserted and packed within the root canal using a measured plugger. The tooth was replanted into its socket and the accurate repositioning was confirmed radiographically (Figure 1D). Histopathologic diagnosis for the lesion was periapical granuloma (Figure 2). The extraoral procedure time was 5 min. The patient was given postoperative instructions for a soft diet and careful oral hygiene. 

The treated tooth was rechecked after 10 days. The clinical signs/symptoms had resolved and the sinus tract had disappeared. The healing process was uneventful; the periodontal examination showed normal sulcular depth and normal gingival status. Clinical examination at the 6-month and 1-year recalls revealed satisfactory clinical function, the absence of pain/tenderness to percussion/palpation, as well as the absence of tooth mobility and signs/symptoms of inflammation or infection. Periapical radiographs revealed healing of the periapical lesion substituted with newly formed bone; external root resorption of involved tooth ceased without radiographic signs of replacement resorption (Figure 1E-F).

## Discussion

Extraoral endodontic treatment (EET) is rewarded by some researchers; and they suggest considering EET as the last option after failure of other endodontic treatments [9]. On the other hand, others recommend EET as an economical and conventional technique within the shorter time and easy manipulation [10-12]. Major concern in extraoral endodontic treatment is the additional damage implemented on the PDL by prolonged extraoral periods and extraoral root-filling material/procedure [13]. 

It is stated that EET has several advantages over endodontic surgery since it is less complicated, protracted, invasive, and expensive [14]. In the present case, all critical parameters were carefully evaluated prior to performing EET; if case selection is carried out correctly, the treatment’s ease and prognosis raise. EET was chosen as the treatment option because of the mid-treatment evaluation and the patient’s refusal for tooth extraction. The 6- and 12-month follow-ups confirmed the successful management of this hopeless tooth. 

Management of IERR is based on removing bacteria and their byproducts from the root canal space as well as dentinal tubules to stop the inflammatory processes and allow healing of periodontal tissues [2]. The conventional and preferred treatment protocol for a progressive IERR comprises of chemo-mechanical preparation of the root canal system by using NaOCl irrigation as well as inter-appointment dressing of the canal with CH to provide an alkaline pH inside the dentinal tubules to eliminate the bacteria and neutralize their endotoxin [4, 15]. However, in the present case, irrigation with full strength NaOCl during cleaning and shaping of the root canal and placement of intracanal medicament did not manage the infection during 1-week post-operation period. Although, bacteria on the surfaces of root canal dentinal walls are more easily killed than those protected in the depths of dentinal tubules, bacteria inside the tubules may be possibly affected by antibacterial components leaching from the endodontic therapeutics [16]; even after these procedures, viable bacteria may still be found inside the dentinal tubules, with the potential for disease to persist or emerge [17].

Surgical endodontic literature supports a direct cause-and-effect relationship between creations of hermetic apical seal by endodontic filling materials and successful outcomes [18]; an ideal root-filling material should be antibacterial, moisture resistant, dimensionally stable, biocompatible, and able to induce regeneration of the PDL, particularly cementogenesis and osteogenesis [6, 7, 19]. 

Sealing ability of CEM cement as root-end filling material is comparable with MTA [6]. An interesting study showed that CEM cement and CH showed similar favorable results against four bacterial species, which was even superior to MTA [20]. Moreover, MTA and CEM cement, as two water-based endodontic biomaterials, did not demonstrate shrinkage upon setting [21]; they form hydroxyapatite crystals over their surfaces [22]. Several *in vivo* studies specifically in the field of regenerative endodontics [23] and vital pulp therapy [24-32], showed that CEM cement has favorable biocompatibility [33, 34]. Furthermore, randomized controlled trials have shown that cementogenic properties of CEM cement are comparable to those of MTA when used for root perforation repair [8] or as a root-end filling material [7].

## Conclusion

Extraoral retrograde root canal filling with CEM cement in a refractory apical lesion caused by orthodontically induced extensive IERR, may be a suitable treatment choice in current practice. 
